# MicroRNAs: Potential Targets for Developing Stress-Tolerant Crops

**DOI:** 10.3390/life11040289

**Published:** 2021-03-28

**Authors:** Saurabh Chaudhary, Atul Grover, Prakash Chand Sharma

**Affiliations:** 1 Cardiff School of Biosciences, Cardiff University, Cardiff CF10 3AT, UK; 2 Defence Institute of Bio-Energy Research, Defence Research and Development Organisation (DRDO), Haldwani 263139, India; atul@diber.drdo.in; 3 University School of Biotechnology, Guru Gobind Singh Indraprastha University, New Delhi 110078, India

**Keywords:** microRNA, abiotic stresses, biotic stresses, crop improvement, NGS, transcriptome

## Abstract

Crop yield is challenged every year worldwide by changing climatic conditions. The forecasted climatic scenario urgently demands stress-tolerant crop varieties to feed the ever-increasing global population. Molecular breeding and genetic engineering approaches have been frequently exploited for developing crops with desired agronomic traits. Recently, microRNAs (miRNAs) have emerged as powerful molecules, which potentially serve as expression markers during stress conditions. The miRNAs are small non-coding endogenous RNAs, usually 20–24 nucleotides long, which mediate post-transcriptional gene silencing and fine-tune the regulation of many abiotic- and biotic-stress responsive genes in plants. The miRNAs usually function by specifically pairing with the target mRNAs, inducing their cleavage or repressing their translation. This review focuses on the exploration of the functional role of miRNAs in regulating plant responses to abiotic and biotic stresses. Moreover, a methodology is also discussed to mine stress-responsive miRNAs from the enormous amount of transcriptome data available in the public domain generated using next-generation sequencing (NGS). Considering the functional role of miRNAs in mediating stress responses, these molecules may be explored as novel targets for engineering stress-tolerant crop varieties.

## 1. Introduction

Increasing global population and livestock demand a substantial increase in the production of food and fodder. According to the United Nations Population Division, the global population will touch the mark of 8.3 billion by 2030. To serve better quality food and feed the ever-growing population is the imperative task for the scientific community in the 21st century. Furthermore, changing climatic conditions adversely affect agricultural productivity worldwide. Extreme climatic conditions are the major cause of abiotic and biotic stresses, and more than 50% crop yield loss per annum worldwide [1]. Therefore, plant biology research activities require the development of high yielding, stress-tolerant crop varieties with desired nutrients to face food security challenges in the coming times. Classical crop breeding has been practiced for hundreds of years to generate high yielding crop varieties, and significant progress has been made to utilize genetic variations available in germplasm resources to develop crops with desirable agronomical traits. However, the long generation time and self-crossing of crops make the classical breeding techniques more time consuming and cumbersome. In that scenario, alternate efficient strategies are required to develop crop varieties with high yield and stress resistance. Genetic engineering is one such strategy that is currently being utilized and practiced worldwide to enhance the yield of crops through the development of environmental stress- and disease-resistant crop varieties [2]. However, since a single trait might be controlled by many genes or vice-versa, the so-called pleiotropic effect makes the agronomical traits genetically complex. Therefore, improving a trait via genetic engineering sometimes may adversely affect other important traits. Moreover, many agronomical traits such as high yield and stress tolerance are regulated by a group of genes or pathways, making selection of gene(s) for desirable trait(s) rather difficult. Thus, the manipulation of agronomical traits to improve crop production requires genetic modulators that act precisely and target in a specific manner.

In the recent past, microRNAs (miRNAs) emerged as a novel target in the field of genetic engineering and have been exploited to develop high yield and stress-tolerant crop varieties [3,4,5,6]. MiRNAs are 20–24 nucleotide long non-coding endogenous regulatory RNAs which regulate many biological processes by gene silencing at the transcriptional and post-transcriptional level [7]. The miRNAs induce gene regulation through pairing and cleavage of their targeted mRNA or by inhibiting protein translation [7]. In plants, primary miRNA (pri-miRNA) is encoded by endogenous miRNA coding genes, transcribed by RNA polymerase II (Pol II). After a series of enzymatic reactions, pri-miRNAs fold into a stem-loop secondary structure to form mature miRNAs, which pair with respective target mRNAs/transcripts to destabilize them or inhibit protein translation [7,8,9].Several studies in the recent past have suggested versatile roles of miRNAs in plants, where they are involved in almost all biological and metabolic processes, including plant growth and development timing, tissue and organ differentiation, plant architecture, organ polarity, and response to various abiotic and biotic stresses [7,10,11,12,13,14]. Moreover, many studies have reported the differential expression of miRNAs and their targeted genes during different stages of plant development and tissue differentiation [15,16,17,18], organ phase transition [16], and under various environmental stresses [4,19,20]. The differential expression of miRNA further helps in the selection and identification of miRNAs and their target genes responsible for agronomical traits of interest.

Evidence has been collected in the recent past from miRNA analysis in various plant species including crops such as rice, maize, wheat, sorghum, sunflower, and cotton under different stresses, suggesting the potential role of miRNAs in regulating stress response in plants [4,11,21,22,23,24,25]. Manipulation of a single miRNA may enhance tolerance to multiple abiotic stresses in plants. For instance, overexpression of miR408 in Arabidopsis enhanced tolerance to salinity, cold, and oxidative stress [26]. Similarly, various miRNAs have been reported with altered expression in response to various fungal and viral infections in crop plants [27,28]. Therefore, plant miRNAs may serve as major candidates for further enhancing our understanding of plant stress responses at the molecular level [3,4]. Understanding plant miRNA regulatory pathways equips us with novel tools for genetic engineering to further improve crop yield, quality, and abiotic and biotic stress tolerance in crop varieties.

## 2. Biogenesis and Mode of Action of Plant miRNAs

**Figure 1:**
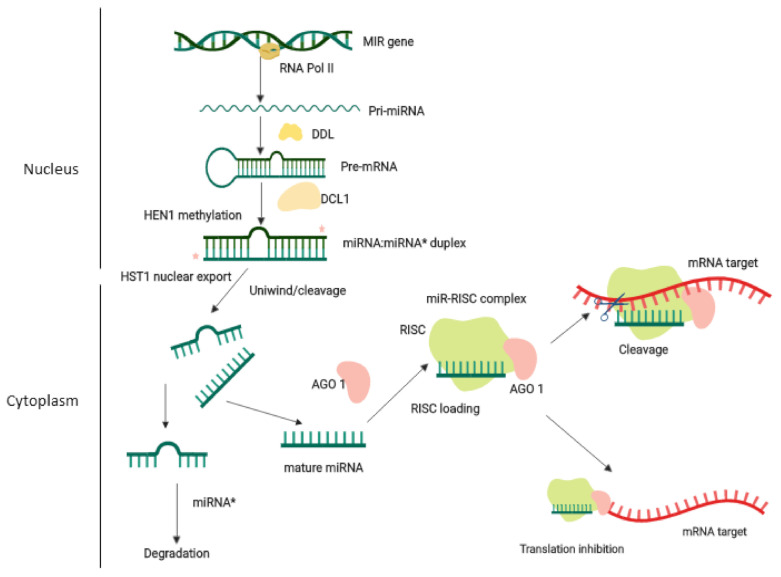
MicroRNA (miRNA) biogenesis and mode of action. Briefly, a miRNA gene (MIR gene), is transcribed into long single stranded preliminary-miRNA (pri-miRNA) transcript with the help of RNA polymerase II (RNA Pol II) in the nucleus. The pri-miRNA is converted into stem loop structure called precursor-miRNA (pre-miRNA), which is stabilized by the DAWDLE (DDL) enzyme. The Dicer-like 1 (DCL1), with the help of other proteins, generates miRNA:miRNA* duplex structure from pre-miRNA. The 3’ ends of miRNA:miRNA* duplex are methylated (stars) by HUA ENHANCER 1 (HEN1) and exported to the cytoplasm with the help of HASTY (HST1) enzyme. In the cytoplasm, the duplex is cleaved into mature miRNA from one strand, and the other strand miRNA* gets degraded. The mature miRNA is further processed by ARGONAUTE 1 (AGO1) and loaded into RNA-induced gene silence complex (RISC) to form miR-RISC complex. Depending upon the complementary sequence of the target mRNA, miR-RISC complex acts either by cleaving target mRNA or by inhibiting its translation. The figure is created with BioRender app (https://app.biorender.com/; accessed on 30 April 2020).

The biogenesis of miRNAs is initiated inside the nucleus. A brief graphical representation of the biogenesis of plant miRNAs is provided in Figure 1. In general, genes encoding plant miRNAs, called microRNA genes (MIR genes), are found in intergenic areas or in antisense/sense orientation within introns of other genes [7]. The MIR genes are transcribed by RNA polymerase II to form a long RNA transcript called pri-miRNA [7]. Like other transcripts, the pri-miRNAs are capped at 5’ end and polyadenylated at 3’ end. The partial sequence of long single stranded pri-miRNA folds into a perfectly stem-loop structure, which is stabilized by RNA-binding protein, DAWDLE (DDL), to form precursor miRNA (pre-miRNA) [7,11]. The one arm of the stem-loop structure of pre-miRNA represents the mature miRNA sequence, which is further recognized by an endoribonuclease called Dicer-like (DCL1), an RNAIII type enzyme, with other proteins such as HYPONASTIC LEAVES 1 (HYL1), and SERRATE (SE) [29,30]. The DCL, HYL1, and SE processed the stem-loop structure of pre-miRNA to generate miRNA:miRNA* duplex structure inside the nucleus [7,29,30,31]. To stabilize and protect this newly synthesized miRNA:miRNA* duplex from degradation, it is methylated at 3’ terminus by a small RNA methyltransferase protein named HUA ENHANCER 1 (HEN1) and exported to the cytoplasm with the help of HASTY (HST1), a plant homolog of animal EXPORTIN-5 [32,33,34,35].

Finally, the mature miRNA in cytoplasm unwinds and is loaded to RNA-induced gene silencing complex (RISC), where it regulates expression of genes by forming the miR-RISC complex. The miR-RISC complex is stabilized by the ARGONAUTE 1 (AGO1) protein. The miRNA:miRNA* duplex then primarily unwinds with the help of AGO1 protein, and one strand is directed to exosomes for degradation, whereas the other strand of mature miRNA remains attached to the RISC with AGO1 protein [36,37,38]. The mature miRNA finally guides the AGO1-containing RISC complex, either to direct site-specific cleavage of complimentary mRNA with high homology or inhibit the translation of the targeted mRNA by imperfect base pairing. Regarding gene regulation by miRNA, the previous assumption suggested that only mature miRNA inhibits the mRNAs and translation. However, recent studies have demonstrated that another miRNA* strand also has its own targeted mRNA and regulates expression of respective genes [39].

In the recent past, there has been an increase in the number of reports investigating the mechanism of miRNA-based regulation of gene expression [5,40,41]. Plant miRNAs generally regulate gene expression at transcriptional and post-transcriptional level through perfect complementary sequence pairing [11,40]. The two modes of mechanism include cleavage of target mRNA, which is the result of a perfect pairing, and translation inhibition, a consequence of imperfect pairing. In the first mode, miRNA cleaves the poly-(A) tail of the target mRNA leading to its destabilization and decay [11,40,42]. Additionally, miRNA helps to influence various biological processes at transcriptional level by silencing transcription activity and decreasing the level of random fluctuation in the transcripts’ copy number [5,43]. Experimental data from overexpression or loss of function analyses suggest that miRNAs fine-tune the expression of diverse class regulatory genes. Approximately 66% and 24.2% of miRNA targets are transcription factors (TFs) and major class of R (resistance) genes suggesting the role of miRNAs in diverse gene regulatory networks and plant immune system [44].

## 3. Mining of miRNAs 

Identification of MIR genes, miRNAs, and their target gene is the foremost step to elucidating the miRNA-mediated gene regulatory network and underlying mechanisms. The initial research on plant miRNA identification included direct cloning and sequencing of small RNA population strategies [33,45]. In the past decade, advancement in high-throughput sequencing, also called next generation sequencing (NGS) technology, and computational strategies, has enhanced the discovery of novel and conserved plant miRNAs dramatically in a tissue-, environment-, and time-specific manner. The NGS technology has revolutionized plant miRNA research by enabling genome-wide or transcriptome-wide identification of miRNAs with unrivalled coverage and depth [5,41,46]. Additionally, high sequence similarity with the target mRNAs and the conserved nature of miRNAs makes computational tools, such as Basic Local Alignment Tool (BLAST) (https://blast.ncbi.nlm.nih.gov/Blast.cgi, accessed on 9 March 2021) and other homology-based tools, an alternative approach for the identification of miRNAs in many crop plants [47,48,49].

### 3.1. Next Generation Sequencing-Based Methods for Identification of miRNAs

NGS technology, such as the RNA-sequencing (RNA-Seq) technique, is widely used for transcriptome profiling and differential gene expression analysis requiring isolation of poly(A)-tail mRNA. The presence of poly-(A) tail in pri-miRNA includes them in the mRNA population used for RNA-Seq. Since pri-miRNAs are not stable enough for sequencing, it is difficult to fish out pre-miRNAs directly from RNA-Seq libraries. However, the robust and high depth of the RNA-Seq technique enables the detection of lowly abundant and weakly expressed transcripts. Therefore, pri-miRNA could be identified in the RNA-Seq data, although the precise level and full length of pri-miRNA could not be predicted in the RNA-Seq data [46]. To overcome the problem of detection of full-length transcripts in RNA-Seq, its modification, called RNA-paired-end tag sequencing (RNA-PET-Seq) [50,51], was evolved. RNA-PET-Seq enables capturing transcripts with 5’ and 3’ ends simultaneously and distinguishes the boundaries of transcription units, providing sufficient information to assemble full length transcripts [50,51]. The combination of RNA-Seq and PET-tags can serve as a high-throughput strategy to elucidate the MIR gene transcriptional regions and quantify the abundance of a pri-miRNA [46]. Another revolutionary modification in the RNA-Seq technique occurred with the introduction of small RNA-Sequencing (sRNA-Seq) [52,53]. Since the library for sRNA-Seq is prepared from small RNA, it enables quantification of the abundance of miRNA in plant tissues, in a condition- and time-dependent manner. Further, techniques such as double-stranded RNA-Sequencing (dsRNA-Seq) and single stranded RNA-Sequencing (ssRNA-Seq) have also been used in the discovery of miRNAs [54]. The dsRNA-Seq and ssRNA-Seq together allow to elucidate the stem loop structure of the pre-miRNA efficiently [46]. Another widely used high-throughput technique in miRNA research is degradome-sequencing (degradome-Seq) [55,56]. The major application of degradome-Seq facilitates the identification of truncated transcripts generated from endonucleolytic cleavages, guided by small RNAs and miRNAs. Therefore, the reads generated from degradome-Seq could provide the information of the slicing sites residue in miRNA specific to their target transcripts [46].

### 3.2. In Silico Mining of miRNAs

**Figure 2:**
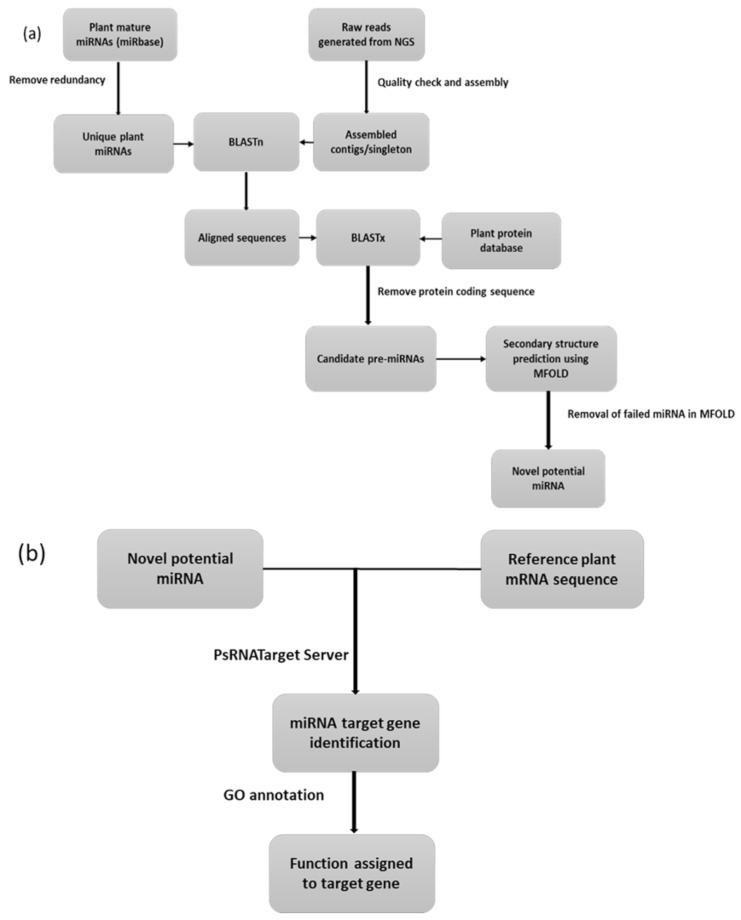
Workflow for in silico mining of (**a**) plant miRNA, and (**b**) target genes from transcriptomic data generated using next generation sequencing (NGS). The flowchart is modified from Panda et al. (2014) [62].

The present-day high-throughput techniques generate a huge amount of data from diverse plant species, including crop plants. All the data so generated have been deposited in public domains such as miRBase (http://www.mirbase.org, accessed on 9 March 2021) [57,58], the Plant MicroRNA Database (PMRD; http://mirnablog.com/plant-micrornadatabase-goes-online, accessed on 9 March 2021) [59], and the NCBI-Gene Expression Omnibus (GEO; http://www.ncbi.nlm.nih.gov/geo/, accessed on 9 March 2021) [60]. Moreover, the conserved nature of plant miRNA [31] provides reasonable results on homology-based in silico analysis of potential miRNAs and their target genes [61]. Additionally, the developments in computational biology approaches also provide significant support to handle a large amount of raw data and make it biologically meaningful. A schematic representation for in silico mining of miRNA is provided in Figure 2. Briefly, the sequence for mature plant miRNA can be downloaded from miRBase (http://www.mirbase.org, accessed on 9 March 2021) [57,58], followed by removal of redundant miRNA sequences. The raw reads generated from NGS can also be downloaded from the public domain, such as NCBI-Gene Expression Omnibus (GEO; http://www.ncbi.nlm.nih.gov/geo/, accessed on 9 March 2021) [60]. The reads after quality filters are assembled into contigs/singletons. The unique contigs/singletons are further subjected to nucleotide BLASTn (https://blast.ncbi.nlm.nih.gov/Blast.cgi, accessed on 9 March 2021) with the unique plant miRNA to get aligned sequences. The candidate miRNA should fulfil the two criteria as described by Panda et al., (2014) [62]: (i) at least 18-nt length with no gap in between should be there in candidate miRNA, and (ii) the assembled sequences, which match closely to the known miRNAs, are to be selected for further study [62]. The aligned sequence is then further put to BLASTx with plant protein database (Uniport; https://www.uniprot.org/program/Plants, accessed on 9 March 2021) [63] to remove all coding sequences. After discarding the protein coding sequence, the secondary structure can be accessed using MFOLD software (http://unafold.rna.albany.edu/?q=mfold, accessed on 9 March 2021) [64] with default parameters. The candidate pre-miRNA, left after discarding miRNA that failed in MFOLD criteria, could be considered as novel potential miRNAs. The novel potential miRNAs can further be used for the identification of their target genes based on complementary binding between miRNA and target gene sequences using the psRNATarget server (http://plantgrn.noble.org/psRNATarget/?dowhat=Help, accessed on 9 March 2021) [65]. Finally, gene ontology (GO) terms using QuickGO (https://www.ebi.ac.uk/QuickGO/, accessed on 9 March 2021) can be assigned to the target genes to validate their functionality. 

## 4. Functional Role of miRNAs in Plant Stresses

Abiotic and biotic stresses have become the major factors in limiting crop productivity. Many studies suggest the important role of plant miRNAs in response to abiotic and biotic stresses [3,4,19,20,66,67]. Several miRNAs show differential expression under different environmental conditions; however, these expression changes depend on many factors such as the type and magnitude of stress, plant species, and miRNA involved [4]. Nevertheless, an appropriate genetic engineering approach needs to be applied to validate the expression and molecular mechanism underlying the response of plant miRNA to stresses.

### 4.1. Role of Plant miRNAs in Abiotic Stress

Plants, during their entire life cycle, encounter several abiotic stresses including drought, salinity, heavy metals, oxidative stress, and extreme temperatures. Abiotic stresses pose serious deleterious implications on plant growth and development caused due to the oxidative damage of lipids, protein, and DNA, as well as the accumulation or abnormal increase in the levels of molecules such as osmolytes (during drought and salinity stress) or reactive oxygen species (in case of oxidative stress) within the plant [24,68,69]. Over the years, miRNA has emerged as an important candidate in managing plants’ responses to abiotic stress [4,12,39]. The first report that provided a direct link between the levels of miRNA and plant stress responses was the miR398 that targets two closely related Cu/Zn superoxide dismutase coding genes (CSD1 and CSD2) and miR395 and miR399, which target the sulfate transporter (AST68) and the phosphate transporter (PHO1), respectively [70]. Later, more and more evidence accumulated showing the aberrant expression of miRNA under abiotic stress in various crop plants, including wheat, maize, rice, cotton, barley, and many others. Table 1 lists the major findings regarding role of miRNA in response to abiotic and biotic stresses in various crop plants. Manipulating a single miRNA in plants significantly changes the stress tolerance capability; thus, among various miRNAs, some are identified as promising targets for developing transgenics with improved abiotic stress tolerance [4,39]. For instance, overexpression of miR169 leads to higher tolerance to water deficiency during early plant development in tomatoes [71]. The transgenic tomatoes developed the ability to retain more water inside the cell and require less water from the soil [71]. The study further showed that overexpression of miR169 causes a reduction in the stomatal aperture index and stomatal conductance thereby significantly reducing the transpiration rate in transgenic tomato. Subsequently, overexpression of miR169, the largest and most conserved family of miRNAs, was validated under different abiotic stresses in several other plant species, including some crops also. For example, overexpression of miR169 led to enhanced tolerance capacity against drought and salinity in *Agrotis stolonifera* (bentgrass) [72]. Moreover, overexpression of miR169 increases cold stress tolerance in rice [73]. In Arabidopsis, overexpression of miR169 makes the plant hypersensitive to nitrogen starvation [74].

The role of other miRNA families viz. miR156, miR159, miR319, miR393, miR394, miR395, miR395, miR396, miR402, miR417, and miR828, in abiotic stress response has also been validated following a transgenic approach in several plant species [4,24]. For example, overexpression of miR156 and miR159 enhanced the heat stress tolerance in Arabidopsis [75] and rice [76], respectively. Similarly, manipulation of miR319 enhances the multiple stress tolerance ability in plants. Over-expressed miR319 increases chilling tolerance in rice [73] as well as drought tolerance in bentgrass [72]. The miR393 and miR396 mediate multiple stresses such as drought, heat, and salinity tolerance capacity in transgenic rice [77,78,79]. Moreover, overexpressed miR828 helps sweet potato to tolerate oxidative stress by exhibiting increased lignin biosynthesis and hydrogen peroxide production [80]. There is a long list of crop plants, which have been genetically engineered to improve abiotic stress tolerance using overexpression or knock out of particular miRNA. The evidence discussed above clearly demonstrate that miRNAs have become the new target for crop improvement and in developing abiotic stress tolerance in crop varieties.

### 4.2. Role of Plant miRNA in Biotic Stress

Like abiotic stresses, biotic stresses also adversely affect crop productivity. Several studies reported differential expression of miRNAs and their target genes in crop plants during the attack of insects, fungi, bacteria, viruses, and nematodes [19,20,66]. During evolution, plants developed several sophisticated mechanisms to fight against biotic agents. The regulation of gene expression and networking systems via miRNA is one such mechanism, which enhances the ability of plants to fight against various pathogens. Several NGS studies conducted in biotic stress environments allowed the identification of miRNAs. For instance, strip virus infection in rice downregulated the expression of miR160, miR166, miR171, and miR396 families [67]. Some of these miRNAs were further employed in genetic engineering to develop biotic stress tolerance in crops. For example, overexpression of miR396 develops more tolerance against fungal infection in transgenic *Medicago truncatula* as compared to wild type plant [67]. In another example, an Arabidopsis mutant repressed for miR159 showed increased tolerance to root knot nematodes [66]. Other miRNA families that have been exploited in genetic engineering approaches against biotic stress include miR160, miR398, miR393, and miR397 in rice [81,82], miR482 in tomato [83], miR396 in tobacco [84], and miR171 in *Medicago truncatula* [85].

**Table 1:** List of studies on the functional role of different miRNA/miRNA families in the regulation of abiotic and biotic tresses in major crop species (List updated January 2011–December 2020).

Crop	MicroRNAs	Stress Responses	Reference
Alfalfa (*Medicago sativa*)	multiple miRNAs	Drought stress	[86]
	miR3512, miR3630, miR5213, miR5294, miR5368 and miR6173	Drought stress	[87]
	miR156	Heat stress	[88]
Apple (*Malus sylvestris*)	multiple miRNAs	Drought stress	[89]
Barley (*Hordeum* L.)	multiple miRNAs	Drought stress	[90]
	Hv-miR827	Drought stress	[91]
	Ath-miR169b, Osa-miR1432, Hv-miRx5, Hv-miR166b/c	Drought stress	[92]
	multiple miRNAs	Drought stress	[93]
	multiple miRNAs	Salt stress	[94]
Bean (*Phaseolus vulgaris*)	multiple miRNAs	Drought stress	[95]
	miR399	Phosphorus deficiency	[96]
Brassica (*Brassica juncea*)	multiple miRNAs	Abiotic stresses	[97]
Brassica (*Brassica napus)*	miR1885	Immune response	[98]
	miR397a, miR397b and miR6034	Various stresses	[99]
	multiple miRNAs	Drought and salt stress	[100]
Broccoli (*Brassica oleracea*)	multiple miRNAs	Heat stress	[101]
Cabbage (*Brassica* L.)	multiple miRNAs	Heat and drought stress	[102]
	multiple miRNAs	Turnip Mosaic Virus infection	[103]
Cassava (*Manihot esculenta*)	miR160, miR393	Anthracnose disease	[104]
Celery (*Apium graveolens*)	multiple miRNAs	Heat and cold stress	[105]
Chickpea (*Cicer arietinum*)	multiple miRNAs	Ascochyta blight disease	[106]
	multiple miRNAs including miR5213, miR5232, miR2111 and miR2118	Wilt and salt stress	[107]
Cotton (*Gossypium* L.)	miR414	Salinity stress	[108]
	ghr-miR399 and ghr-156e	Salt stress	[94]
	miR319	Abiotic stress signaling	[109]
	ghr-miR5272a	Immune response	[110]
	multiple miRNAs	Salt stress	[111]
	multiple miRNAs	High temperature	[112]
	multiple miRNAs	Low and high temperature stress	[113]
	miR156a/d/e, miR167a, miR169, miR397a/b, miR399a, miR535a/b, miR827b,	Salt stress	[114]
Cowpea (*Vigna unguiculata*)	multiple miRNAs	Drought stress	[115]
Date Palm (*Phoenix dactylifera*)	multiple miRNAs	Salinity stress	[116]
Flax ( *Linum usitatissimum*)	miR319, miR390, and miR393	Aluminum stress	[117]
Foxtail Millet (*Setaria italica*)	multiple miRNAs	Drought stress	[118]
	multiple miRNAs	Dehydration stress	[119]
Java waterdropwort (*Oenanthe javanica*)	multiple miRNAs	Various abiotic stress	[120]
Maize (*Zea mays*)	multiple miRNAs	Chilling stress	[121]
	multiple miRNAs	Heat stress	[122]
	multiple miRNAs	Nitrogen stress	[123]
	multiple miRNAs	Drought stress	[82]
	multiple miRNAs	Cadmium stress	[124]
	multiple miRNAs	Phosphate deficiency	[125]
	multiple miRNAs	Water logging	[126]
	multiple miRNAs	Nitrogen deficiency	[127]
	multiple miRNAs	Short term water logging	[128]
	miR160, miR164, miR167, miR168, miR169, miR172, miR169, miR395, miR397, miR398, miR399, miR408, miR528, miR827	Low nitrate availability	[129]
Peach (*Prunus persica*)	multiple miRNAs	UVB radiations response	[130]
Pear (*Pyrus pyrifolia*)	multiple miRNAs	Apple stem grooving virus infection and high temperature	[131]
Potato (*Solanum tuberosum*)	multiple miRNAs	Nitrogen stress	[132]
	Stu-mi164	Osmotic stress	[133]
	miR172, miR396a, miR396c, miR4233, miR2673, miR6461	Drought stress	[134]
Radish (*Raphanus sativus*)	ath-miR159b-3p, athmiR159c, ath-miR398a-3p, athmiR398b-3p, ath-miR165a-5p, ath-miR169g-3p, novel_86, novel_107, novel_21, ath-miR171b-3p	Heat stress	[135]
	multiple miRNAs	Cadmium stress	[136]
	multiple miRNAs	Chromium stress	[137]
	multiple miRNAs	Salt stress	[138]
	multiple miRNAs	Cadmium stress	[139]
Rice (*Oryza sativa*)	miR408, miR528	Cadmium stress	[140]
	multiple miRNAs	Arsenic stress	[141]
	multiple miRNAs	High temperature and salt stress	[142]
	multiple miRNAs	Cold stress	[143]
	miR169, osa-miR444a.4-3p	Nitrogen starvation	[144]
	miR529a	Oxidative stress	[145]
	miR393, miR390	Multiple stress	[146]
	Osa-miR820	Salt stress	[147]
	multiple miRNAs	Phosphate Starvation	[148]
	miR399, miR530	Nitrogen starvation	[149]
	miR156, miR164, miR167, miR168, miR528, miR820, miR821, miR1318	Low-nitrogen stress	[150]
	multiple miRNAs	Abiotic stress	[151]
	osa-miR414, osa-miR164e, osa-miR408	Salt stress	[152]
Soybean (*Glycine max*)	multiple miRNAs	Water deficit	[153]
Sugarcane (*Saccharum* L.)	multiple miRNAs	Water-deficit stress	[154]
	multiple miRNAs	Low temperature stress	[155]
	multiple miRNAs	Waterlogging condition	[156]
	multiple miRNAs	Drought stress	[157]
	multiple miRNAs	Drought stress	[158]
Sweet Potato (*Ipomoea batatas*)	multiple miRNAs	Drought and CO_2_ stress	[159]
	multiple miRNAs	Salt stress	[160]
Switchgrass (*Panicum virgatum*)	multiple miRNAs	Drought and heat stress	[161]
	multiple miRNAs	Salt stress	[162]
Tobacco (*Nicotiana tabacum*)	multiple miRNAs	Salt and alkali stress	[163]
Tomato (*Solanum lycopersicum*)	multiple miRNAs	Drought and heat stress	[164]
	multiple miRNAs	Drought stress	[165]
Turnip (*Brassica rapa*)	miR166h-3p-1, miR398b-3p, miR398b-3p-1, miR408d, miR156a-5p, miR396h, miR845a-1, miR166u, Bra-novel-miR3153-5p and Bra-novel-miR3172-5p	Cold stress	[166]
Wheat (*Triticum aestivum*)	multiple miRNAs	Reactive oxygen species (ROS) response	[167]
	multiple miRNAs	Water deficit and heat stress	[168]
	TaemiR408	Phosphate deprivation and salt stress	[169]
	TamiR1139	Phosphate starvation	[170]
	multiple miRNAs	Cold stress	[171]
	multiple miRNAs	Drought stress	[172]
	miR159, miR160, miR166, miR169, miR172, miR395, miR396, miR408, miR472, miR477, miR482, miR1858, miR2118, miR5049	Drought stress	[173]
	multiple miRNAs including miR159, miR393, miR398	Cold, wound, and salt stress	[174]
	Tae-miR408	Salinity, cupric metal, and stripe rust stress	[175]

## 5. Current miRNA-Based Strategies for Crop Improvement

**Figure 3:**
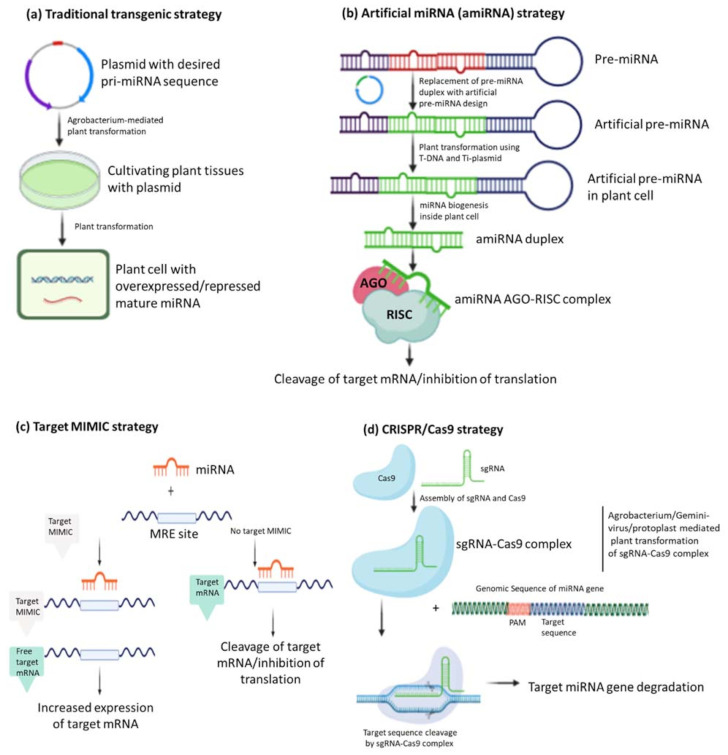
Overview of miRNA-based strategies for crop improvement. Illustrating (**a**) the traditional transgenic approach targeting directly primary-miRNA (pri-miRNA) in plants; (**b**) the artificial miRNA (amiRNA) strategy to enhance or repress miRNA expression in plants (Sablok et al., 2011). The amiRNA is designed to have a complementary sequence to the target mRNA and stem-loop structure like the original miRNA. The amiRNA then transfers into the plant cell using traditional transformation techniques, where its biogenesis occurs like original miRNA. Finally, amiRNA targets the mRNA without affecting non-target genes; (**c**) target MIMIC strategy where target MIMIC instead of target mRNA is recognized by miRNA; (MRE site: miRNA recognition site) [194]; (**d**) miRNA-targeting CRISPR/Cas9 approach to manipulate the miRNA gene using sgRNA-Cas9 complex. CRISPR/Cas9 techniques based on two components, (i) sgRNA: single guide RNA, and (ii) Cas9 endonucleases. The sgRNA consists of a 20-nt-long spacer sequence which is highly specific to target DNA having a 5’-NGG-3’PAM (protospacer adjacent motif). The Cas9 vector construct and sgRNA complex transfer into a plant cell using a transformation technique. In the plant cell, sgRNA-Cas9 complex target and cleave the DNA and degrade the targeted gene. This figure was created with the BioRender app (https://app.biorender.com/; accessed on 30 April 2020).

Several miRNA-based strategies are currently being exploited in the field of crop improvement. Genetic tools such as high throughput sequencing, quantitative-real time polymerase chain reaction (qRT-PCR), and other gene expression analytic tools are used to elucidate the functional role of plant miRNAs. However, these tools do not provide any direct evidence of gene functionality but are utilized for the identification and re-validation of the related function of plant miRNAs. Another strategy utilized for exploring miRNA function in crop improvement is the traditional transgenic approach. Earlier, many studies recorded the overexpression or repression changes in the miRNA and related gene function in transgenic plants. However, since a single miRNA may regulate several genes, its overexpression or repression sometimes produces undesirable phenotypic changes also. Furthermore, over-abundance of miRNA may alter the expression of respective target genes having different roles in plant development, resulting in a deleterious effect on the host plant. Therefore, the implementation of target specific genetic engineering is required for miRNA-based strategies of crop improvement. In the recent past, target specific approaches such as the use of specific promoters rather than whole genes have enabled miRNA-based strategies to be more precise to introduce desirable traits in crop plants. The various miRNA-based strategies currently utilizing for crop improvement is presented in Figure 3.

### 5.1. Traditional Transgenic Strategy

Earlier plant science researchers overexpressed plant miRNA in several crop and model plants to study the role of miRNA and its related gene function. However, the small size of plant miRNA and the requirement of the exact miRNA sequence makes it rather difficult to manipulate. Therefore, following an alternative approach, plant scientists started transferring the long pri-miRNA sequence instead of mature miRNA. This strategy helped in expressing direct mature miRNA rather than manipulating the MIR gene. Further, pre-miRNA from model plant species can be utilized easily for crop plants with unknown genetic information [39]. For instance, the miR156 gene from Arabidopsis can be transferred to eggplants with unknown genetic information to study the function of this miRNA [39]. 

### 5.2. Artificial miRNA (amiRNA) Strategy

To overcome the problem of affecting non-target gene in the plant miRNA traditional transgenic approach, alternative artificial miRNA (amiRNA) was developed [176,177]. The amiRNA approach produces miRNAs and specifically silences the target genes without interfering with the function of other genes [177]. In the amiRNA approach, the gene sequence can be utilized to construct mature amiRNA having the conserved stem-loop structure like original pre-miRNA and complementary sequence to target mRNA. The artificial miRNA:miRNA* duplex can be inserted into the transgenic plant directly in the stem-loop structure to target specific mRNA. In this way, amiRNA can be transferred to target mRNA with high specificity without effecting a non-target gene function as compared to the traditional transgenic approach. An amiRNA has also been utilized in many studies, including knocking out genes for phytopathogens in Arabidopsis and tobacco [178,179,180,181].

### 5.3. Short Tandem Target MIMIC (STTM) Strategy

Like overexpression of plant miRNA using the amiRNA approach, another artificial technique called short tandem target MIMIC (STTM) that modulates the accumulation of miRNA and controls related biological processes was developed. The SSTM, by inhibiting specific miRNA activity, has been employed in several plant species [182,183]. In the STTM strategy, either engineered long non-coding RNA (lncRNA) or circular RNA (circRNA), also called miRNA recognition elements (MRE) with high sequence similarity with target miRNA is transferred to the transgenic plant [184]. This engineered lncRNA or circRNA has two or more conserved binding sites with target miRNA and minor differences in sequences at the cleavage site. This prevents its miRNA cleavage, which remains hybridized but biologically inactive [184,185]. Recently, several MIR genes have been targeted by the STTM approach in crop plants to explore the function of miRNAs [83,92,93]. For example, the function of 35 miRNA families related to important agronomical traits has been studied using STTM strategy in rice [183]. Like STTM, another artificial transcript called miRNA SPONGES having multiple miRNA binding sites, was also engineered in some plant species [184,186]. These miRNA SPONGES are sometimes utilized to inhibit the function of the whole plant miRNA family [83,183].

### 5.4. Clustered Regularly Interspaced Short Palindromic Repeats/CRISPR Associated Gene 9 (CRISPR/Cas 9) Approach

Plant miRNAs have more than one member in a family, and each member plays an important function in a group or individually. The above-mentioned miRNA-based strategies target miRNA without differentiating among members of the miRNA family. To elucidate the role of individual miRNA from a miRNA family, recently developed clustered regularly interspaced short palindromic repeats/CRISPR associated gene 9 (CRISPR/Cas 9) [187,188] proved to be a powerful tool. In protein coding genes, CRISPR/Cas 9 deletes a few nucleotides adjacent to the protospacer motif (PAM) sequence resulting in a frameshift and finally gene silencing. However, the removal of a few nucleotides in miRNA does not efficiently silence MIR genes, which makes it challenging to apply CRISPR/Cas 9. Therefore, only a small number of studies have been reported for the successful implementation of the CRISPR/Cas 9 approach for knocking out miRNA genes [189,190]. For example, miR1514 and miR1509 have successfully been targeted in soybean by CRISPR/Cas 9 [191]. The miRNA1514 and miRNA1509 were targeted using biolistic delivery of a CRISPR/Cas 9 vector for the transient expression [192]. Likewise, in rice, a specific mutation has been induced in miRNA156 recognition sites of the ipa1 gene using CRISPR/Cas 9 to improve the number of traits related to plant architecture [102]. In another report, mono-allelic and bi-allelic mutations in several miRNA genes of the T0 line of rice have successfully been incorporated using CRISPR/Cas 9, resulting in the loss of function of miRNA [193]. Though the genome editing approach has been successfully implemented in some miRNA studies, there are still some gaps needing improvement to thoroughly amend CRISPR/Cas 9 technology for miRNA-based crop improvement.

## 6. Conclusions

In addition to the fundamental role of gene silencing, plant miRNAs play diverse roles in almost all biological (molecular) networks. The potential of plant miRNAs in regulating stress-responsive genes makes them a suitable candidate for developing stress-tolerant crop varieties. A deeper understanding of the molecular mechanism regulated by miRNA in the complex molecular networking systems would enable agricultural scientists to manipulate specific agronomical traits in crops. However, the regulation of multiple genes and networks by single miRNA in plants makes the selection of candidate miRNA to target specific agronomically important trait challenging for the scientists. For such traits, efficient tools are required to decipher pri-miRNA-mediated regulatory networks. Undoubtedly, miRNA-based approaches have huge potential for crop improvement to motivate future inter-disciplinary collaborations between scientists of different expertise. For example, a successful miRNA-mediated genome editing effort requires active collaborative efforts from molecular biologists, geneticists, genome editors, miRNA scientists, and plant breeders. Furthermore, appropriate laboratory experiments and confined field trials are required before realizing the actual potential of miRNA-based genome editing in the field of agriculture. Nevertheless, it is also pertinent to be aware of unwanted side effects arising while using genetic modification approaches using miRNAs in the future. 

## Data Availability

Not applicable.
